# Supplementary material for: A national cross-sectional study of the role of clinician specialty and facility complexity on glucocorticoid prescribing in Veterans
Source: Commun Med (Lond). 2025 May 18;5:184. doi: 10.1038/s43856-025-00869-9 (PMC12086179; doi:10.1038/s43856-025-00869-9)
Supplement: Supplementary file 1 — Supplementary Information [file 43856_2025_869_MOESM1_ESM.pdf]

Supplementary Table 1: Definition of glucocorticoid

Any oral drug formulation dispensed during the study period that:<br>1. Is included in VA class HS051 in the VA National Formulary, and/or<br>2. Has one of the following drug names: prednisone, prednisolone, dexamethasone, triamcinolone, methylprednisolone, betamethasone, hydrocortisone, cortisone

Supplementary Table 2: Rates of glucocorticoid use and prescribing by geographic region

Region	Mean (SD) glucocorticoid use percentage	Mean (SD) prolonged use percentage	Mean (SD) glucocorticoid prescribing percentage
**Continental**	11.8 (2.8)	1.9 (0.5)	18.5 (2.4)
**Midwest**	12.8 (2.9)	2.4 (0.4)	18.0 (3.1)
**North Atlantic**	12.7 (4.5)	2.2 (0.5)	15.2 (2.6)
**Pacific**	9.7 (3.1)	1.9 (0.4)	17.1 (3.0)
**Southeast**	1.3 (2.2)	1.8 (0.3)	19.6 (4.3)
