# Supplementary material for: A national cross-sectional study of the role of clinician specialty and facility complexity on glucocorticoid prescribing in Veterans
Source: Commun Med (Lond). 2025 May 18;5:184. doi: 10.1038/s43856-025-00869-9 (PMC12086179; doi:10.1038/s43856-025-00869-9)
Supplement: Supplementary file 2 — Description of Additional Supplementary Materials [file 43856_2025_869_MOESM2_ESM.pdf]

Description of Additional Supplementary Files

File name: Supplementary Data 1

Description: Specialty designations

File name: Supplementary Data 2

Description: Rates of glucocorticoid prescribing by specialty

File name: Supplementary Data 3

Description: Deciles of glucocorticoid treatmen

File name: Supplementary Data 4

Description: Data file for figure 1a

File name: Supplementary Data 5

Description: Data file for figure 1b

File name: Supplementary Data 6

Description: Data file for figure 2

File name: Supplementary Data 7

Description: Data file for figure 3
