# Supplementary material for: A national cross-sectional study of the role of clinician specialty and facility complexity on glucocorticoid prescribing in Veterans
Source: Commun Med (Lond). 2025 May 18;5:184. doi: 10.1038/s43856-025-00869-9 (PMC12086179; doi:10.1038/s43856-025-00869-9)
Supplement: Supplementary file 10 — REPORTING SUMMARY [file 43856_2025_869_MOESM10_ESM.pdf]

nature portfolio

Corresponding author(s): Beth Wallace
Last updated by author(s): Feb 21, 2025

Reporting Summary

Nature Portfolio wishes to improve the reproducibility of the work that we publish. This form provides structure for consistency and transparency in reporting. For further information on Nature Portfolio policies, see our Editorial Policies and the Editorial Policy Checklist.

Statistics

For all statistical analyses, confirm that the following items are present in the figure legend, table legend, main text, or Methods section.

n/a	Confirmed	
☐	☒	The exact sample size (*n*) for each experimental group/condition, given as a discrete number and unit of measurement
☐	☒	A statement on whether measurements were taken from distinct samples or whether the same sample was measured repeatedly
☐	☒	The statistical test(s) used AND whether they are one- or two-sided<br>*Only common tests should be described solely by name; describe more complex techniques in the Methods section.*
☒	☐	A description of all covariates tested
☒	☐	A description of any assumptions or corrections, such as tests of normality and adjustment for multiple comparisons
☐	☒	A full description of the statistical parameters including central tendency (e.g. means) or other basic estimates (e.g. regression coefficient) AND variation (e.g. standard deviation) or associated estimates of uncertainty (e.g. confidence intervals)
☐	☒	For null hypothesis testing, the test statistic (e.g. *F*, *t*, *r*) with confidence intervals, effect sizes, degrees of freedom and *P* value noted<br>*Give P values as exact values whenever suitable.*
☒	☐	For Bayesian analysis, information on the choice of priors and Markov chain Monte Carlo settings
☒	☐	For hierarchical and complex designs, identification of the appropriate level for tests and full reporting of outcomes
☒	☐	Estimates of effect sizes (e.g. Cohen's *d*, Pearson's *r*), indicating how they were calculated

Our web collection on statistics for biologists contains articles on many of the points above.

Software and code

Policy information about availability of computer code

Data collection	Data were obtained from the Veterans Health Administration Corporate Data Warehouse, using SAS, Version 9.4, of the SAS Enterprise Guide for LINUX (SAS Institute Inc., Cary, NC).
Data analysis	Data analysis was performed using R version 4.4.0.

For manuscripts utilizing custom algorithms or software that are central to the research but not yet described in published literature, software must be made available to editors and reviewers. We strongly encourage code deposition in a community repository (e.g. GitHub). See the Nature Portfolio guidelines for submitting code & software for further information.

Data

Policy information about availability of data

All manuscripts must include a data availability statement. This statement should provide the following information, where applicable:

- Accession codes, unique identifiers, or web links for publicly available datasets
- A description of any restrictions on data availability
- For clinical datasets or third party data, please ensure that the statement adheres to our policy

Data availability: This publication uses, in compliance with local and federal regulatory and legal frameworks, administrative data derived from the medical records of Veterans receiving care at federally funded Veterans Health Affairs medical centers. The Department of Veterans Affairs prevents dataset disclosures to other entities without (1) a data transfer agreement, (2) deidentification of the data set, and (3) appropriate institutional review board approvals. Investigators wishing to

obtain these data should contact the corresponding author to discuss the request.

The numerical results underlying the graphs and charts presented in the main figures are made available as Supplementary Data in Excel format, submitted with our manuscript.

Human research participants

Policy information about studies involving human research participants and Sex and Gender in Research.

Reporting on sex and gender	Summary data on patient sex are reported in table 1. No sex-based analyses were performed. Data on gender were not available.
Population characteristics	This is a descriptive analysis of secondary data. See below for inclusion and exclusion criteria.
Recruitment	This study utilized secondary data derived from claims; no patient recruitment was performed.
Ethics oversight	This study was approved by the VA Ann Arbor Institutional Review Board prior to data analysis.

Note that full information on the approval of the study protocol must also be provided in the manuscript.

Field-specific reporting

Please select the one below that is the best fit for your research. If you are not sure, read the appropriate sections before making your selection.

☒ Life sciences ☐ Behavioural & social sciences ☐ Ecological, evolutionary & environmental sciences

For a reference copy of the document with all sections, see nature.com/documents/nr-reporting-summary-flat.pdf

Life sciences study design

All studies must disclose on these points even when the disclosure is negative.

Sample size	All US Veterans ≥18 years with ≥1 outpatient medical claim and ≥1 outpatient pharmacy claim at a Veterans Health Administration facility between 2021-2022
Data exclusions	Veterans a) without a VHA primary care provider (PCP), as they were likely to receive a substantial portion of medical care in civilian health systems; b) with PCPs located at a facility (N = 5) who transitioned to the Cerner electronic medical record system during 2021 or 2022, as data post-transition is unavailable in the Corporate Data Warehouse (CDW) claims database.
Replication	This is a descriptive analysis of secondary data, thus no replication was persued
Randomization	This is a descriptive analysis of secondary data, thus no randomization was required.
Blinding	This is a descriptive analysis of secondary data, thus no blinding was required.

Reporting for specific materials, systems and methods

We require information from authors about some types of materials, experimental systems and methods used in many studies. Here, indicate whether each material, system or method listed is relevant to your study. If you are not sure if a list item applies to your research, read the appropriate section before selecting a response.

Materials & experimental systems

n/a	Involved in the study	
☒	☐	Antibodies
☒	☐	Eukaryotic cell lines
☒	☐	Palaeontology and archaeology
☒	☐	Animals and other organisms
☐	☒	Clinical data
☒	☐	Dual use research of concern

Methods

n/a	Involved in the study	
☒	☐	ChIP-seq
☒	☐	Flow cytometry
☒	☐	MRI-based neuroimaging

Clinical data

Policy information about clinical studies
All manuscripts should comply with the ICMJE guidelines for publication of clinical research and a completed CONSORT checklist must be included with all submissions.

Clinical trial registration	n/a
Study protocol	This is a descriptive analysis of secondary data, thus no study protocol was required.
Data collection	Data were obtained from the Veterans' Affairs Corporate Data Warehouse, and cleaned and analyzed at the VA Ann Arbor Center for Clinical Management Research, between 1/2020 and 5/2024
Outcomes	No modeling was performed
